# Hepatic Dystrophic Calcification Secondary to Transarterial Chemoembolization: Case Report and Review of Literature

**DOI:** 10.7759/cureus.35765

**Published:** 2023-03-04

**Authors:** Vikash Kumar, Mili Shah, Dhir Gala, Manmeet K Singh, Herby Jeanty, Reeny Thomas, Arnold N Forlemu, Vijay Reddy Gayam, Denzil Etienne

**Affiliations:** 1 Internal Medicine, The Brooklyn Hospital Center, New York, USA; 2 Internal Medicine, American University of the Caribbean School of Medicine, Sint Maarten, SXM; 3 Internal Medicine, St George's University, Grenada, GRD; 4 Gastroenterology and Hepatology, The Brooklyn Hospital Center, New York, USA

**Keywords:** transarterial chemoembolization adverse event, hepatic calcification, calcification, transarterial chemoembolization, hepatocellular carcinoma

## Abstract

Hepatocellular carcinoma (HCC) is a common malignancy usually treated with surgery. Patients who are not suitable for surgery undergo transarterial chemoembolization (TACE) which involves injecting anti-cancer drugs and embolizing agents into the hepatic artery. Although it is a relatively safe procedure with minor side effects, TACE can rarely cause dystrophic calcification in the liver. We report a case of a 58-year-old female who presented with right-sided chest pain. The patient had been previously treated for HCC with a TACE procedure. A chest x-ray revealed hepatic calcification which was likely secondary to the prior TACE. This case study emphasizes the significance of considering TACE as a potential cause of hepatic dystrophic calcification.

## Introduction

Hepatocellular carcinoma (HCC) is a common malignancy and is associated with high mortality. The mainstay of treatment is surgery with the intent to resect the entire tumor by either liver resection, ablation, or transplantation [1]. This treatment is only effective in patients presenting early in the course of their disease without metastatic spread. The majority of the patients with HCC present with advanced tumors and thus, have unresectable tumors [2]. Transarterial chemoembolization (TACE), is the treatment of choice in patients who are poor surgical candidates. 

TACE is a procedure with intraarterial injection of anti-cancer drugs and embolizing agents. TACE can potentially shrink the tumor making it resectable, or eligible for liver transplantation [3]. TACE is considered a relatively safe procedure with common adverse effects such as fever, right upper quadrant pain, nausea, and transaminitis [4]. Hepatic failure, cholecystitis, pancreatitis, common bile duct stricture, tumor lysis syndrome, and pulmonary and cerebral embolism are additional less frequent side effects following TACE [5]. Hepatic calcification post-TACE is a rare complication without a reported incidence or prevalence in the literature. We present a case of an 85-year-old female diagnosed with dystrophic hepatic calcifications after TACE.

This case report was presented as an abstract at the 2023 American College of Gastroenterology Conference on 26th October 2022.

## Case presentation

A 58-year-old female presented to the outpatient gastroenterology clinic. Her past medical history was significant for compensated cirrhosis secondary to hepatitis C virus (HCV) infection and sustained virologic response (SVR). The patient denied any chest pain, shortness of breath, nausea, vomiting, or changes in bowel movement. At presentation, her vital signs were stable and within normal limits. Her physical exam was benign with a non-tender, non-distended abdomen, and normal bowel sounds. Her routine blood tests including complete blood count, basic metabolic panel, and liver function tests were unremarkable. Her serum Alpha-Fetoprotein (AFP) level was sent as a screening protocol which was found to have elevated 464 ng/ml. Magnetic resonance imaging (MRI) of the abdomen was performed which showed a 1.7x 1.6 x 1.4 cm lesion in segments of the liver, adjacent to the bifurcation of the right hepatic vein compatible with HCC (Liver Reporting & Data System (LI-RADS) 5). An LI-RADS score of 5 means that the radiologist is certain that this lesion is HCC. A follow-up computed tomography (CT) scan of the chest and pelvis showed no signs of metastatic disease. After being evaluated by the oncology team, the patient was treated with transarterial chemoembolization with radiofrequency ablation (RFA) for the treatment of the early stage of HCC by the interventional radiologist team.

**Figure 1:**
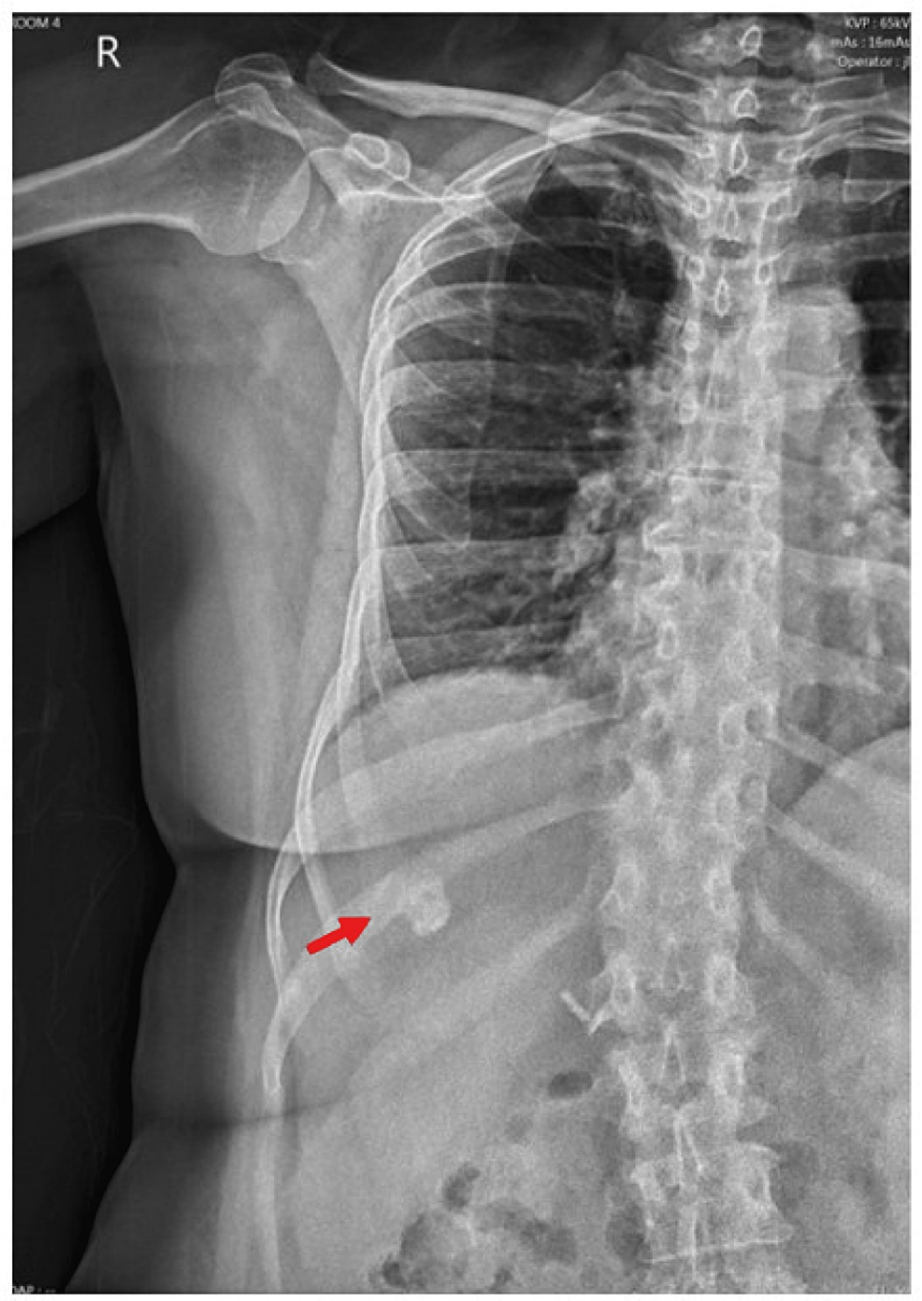
Chest x-ray showing calcific density in the right upper quadrant of the abdomen (red arrow). The calcification is about 1 centimeter in size.

**Figure 2:**
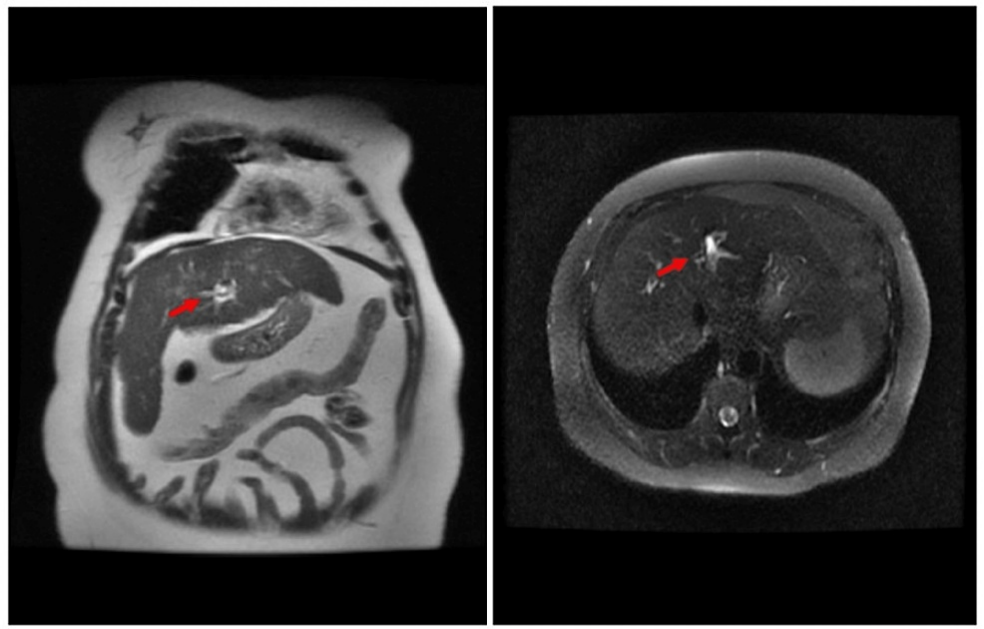
A repeat abdominal MRI revealed no signs of HCC and a stable necrotic treatment cavity with a non-enhancing nodular focus of fat along the posterior aspect of the cavity.

Six months later, the patient presented with chronic intermittent right-sided chest and right-sided abdominal pain associated with early satiety which she started noticing after her TACE procedure. Her vital signs were stable and within normal limits. She had normal physical examination findings. A one-centimeter calcific density of unknown etiology projecting over the right upper quadrant of the abdomen was seen on the chest X-ray (Figure 1). A repeat MRI of the abdomen with and without intravenous (IV) contrast showed a stable 3.9 cm 100% necrotic treatment cavity in the 5/8 segment and a non-enhancing nodular focus of fat along the posterior aspect of the cavity without evidence of new HCC (Figure 2). A radiologist who reviewed the case concluded that the chest x-ray calcification was most likely caused by liver-directed therapy as a result of dystrophic calcification. The patient experienced symptomatic improvement and was managed conservatively with pain medication.

## Discussion

HCC is a common primary malignancy with an annual incidence of 600,000 and is associated with high mortality. In patients presenting early in the course of their disease without metastatic spread, the mainstay of treatment is surgery with the intent to resect the entire tumor by liver resection, ablation, or transplantation [6]. However, the majority of the patients with HCC present later in the disease course with metastatic tumors and thus, are poor surgical candidates. In these patients, TACE is the treatment of choice [7]. TACE involves injecting chemotherapy and embolizing agents intraarterially targeted at the tumor. TACE may be able to reduce the tumor's size, making it amenable to surgery, liver transplantation, or salvage resection [8].

TACE is a relatively safe procedure with minimal complications. The most common adverse effects include fever, right upper quadrant pain, nausea, and transaminitis. Hepatic failure, cholecystitis, pancreatitis, common bile duct stricture, tumor lysis syndrome, and pulmonary and cerebral embolism are additional less frequent side effects following TACE [5]. Among these complications, hepatic calcification post-TACE is a rare event [9].

The pathogenesis of hepatic dystrophic calcification post-TACE is not certain. However, one hypothesis is that the chemotherapeutic agent leads to the precipitation of calcium deposits. This is thought to be secondary to the local inflammation and oxidative stress from the chemotherapy. Additionally, the therapeutic effect of TACE is another potential cause of hepatic calcification. TACE leads to the necrosis of the tumor tissue which can release calcium and other minerals into the bloodstream in high concentrations [10]. 

The diagnosis of hepatic dystrophic calcification is usually made by some form of imaging such as computed tomography (CT) or magnetic resonance imaging (MRI) scan. It usually shows up as punctate, irregular, or diffuse calcification, and may be associated with perihepatic fat stranding in imaging studies. Dystrophic calcification can be differentiated from neoplasia based on its location, shape, and distribution [11].

There are no guidelines for the treatment of hepatic dystrophic calcification after TACE. The most used approach is observation due to the fact that the majority of cases are asymptomatic. In patients with debilitating symptoms such as pain, abdominal distension, or jaundice, surgical intervention may be recommended. 

## Conclusions

The most common adverse effect of TACE is postembolization syndrome presenting with abdominal pain, fever, and nausea hours to days after the procedure. However, patients presenting with persistent abdominal, or chest pain need to be evaluated with CT or MRI which can show dystrophic calcifications. Similar calcifications can be seen with tumor necrosis even in the absence of any therapy however, this is rare. Our case demonstrated a calcific density that was secondary to TACE rather than the recurrence of malignancy. The calcification is secondary to tumor necrosis in response to TACE. This case highlights the importance to consider TACE-related calcification as the differential diagnosis of post-TACE chest or abdominal pain in addition to post-embolization syndrome. 
